# Association of Cardiovascular Disease and Long-Term Exposure to Fine Particulate Matter (PM_2.5_) in the Southeastern United States

**DOI:** 10.3390/atmos12080947

**Published:** 2021-07-23

**Authors:** R. Burciaga Valdez, Mohammad Z. Al-Hamdan, Mohammad Tabatabai, Darryl B. Hood, Wansoo Im, Derek Wilus, Amruta Nori-Sarma, Aramandla Ramesh, Macarius M. Donneyong, Michael A. Langston, Charles P. Mouton, Paul D. Juárez

**Affiliations:** 1 Department of Economics, University of New Mexico, Albuquerque, NM 87131, USA; 2 National Center for Computational Hydroscience and Engineering (NCCHE), Department of Geology and Geological Engineering, University of Mississippi, Oxford, MS 38655, USA; 3 School of Graduate Studies and Research, Meharry Medical College, Nashville, TN 37208, USA; 4 Division of Environmental Health Sciences, College of Public Health, The Ohio State University, Columbus, OH 43210, USA; 5 Department of Family & Community Medicine, Meharry Medical College, Nashville, TN 37208, USA; 6 Department of Environmental Health, School of Public Health, Boston University, Boston, MA 02118, USA; 7 Department of Biochemistry, Cancer Biology, Neuroscience & Pharmacology, Meharry Medical College, Nashville, TN 37208, USA; 8 Division of Outcomes & Translational Sciences, College of Pharmacy, The Ohio State University, Columbus, OH 43210, USA; 9 Department of Electrical Engineering and Computer Science, University of Tennessee, Knoxville, TN 37996, USA; 10 Department of Family Medicine, University of Texas Medical Branch, Galveston, TX 77555, USA

**Keywords:** stroke, diabetes, heart attack, PM_2.5_, health disparities, cardiovascular disease

## Abstract

There is a well-documented association between ambient fine particulate matter air pollution (PM_2.5_) and cardiovascular disease (CVD) morbidity and mortality. Exposure to PM_2.5_ can cause premature death and harmful and chronic health effects such as heart attack, diabetes, and stroke. The Environmental Protection Agency sets annual PM_2.5_ standards to reduce these negative health effects. Currently above an annual average level of 12.0 μg/m is considered unhealthy. Methods. We examined the association of long-term exposure to PM_2.5_ and CVD in a cohort of 44,610 individuals who resided in 12 states recruited into the Southern Community Cohort Study (SCCS). The SCCS was designed to recruit Black and White participants who received care from Federally Qualified Health Centers; hence, they represent vulnerable individuals from low-income families across this vast region. This study tests whether SCCS participants who lived in locations exposed to elevated ambient levels of PM_2.5_ concentrations were more likely to report a history of CVD at enrollment (2002–2009). Remotely sensed satellite data integrated with ground monitoring data provide an assessment of the average annual PM_2.5_ in urban and rural locations where the SCCS participants resided. We used multilevel logistic regression to estimate the associations between self-reported CVD and exposure to elevated ambient levels of PM_2.5_. Results. We found a 13.4 percent increase in the odds of reported CVD with exposure to unhealthy levels of PM_2.5_ exposure at enrollment. The SCCS participants with medical histories of hypertension, hypercholesterolemia, and smoking had, overall, 385 percent higher odds of reported CVD than those without these clinical risk factors. Additionally, Black participants were more likely to live in locations with higher ambient PM_2.5_ concentrations and report high levels of clinical risk factors, thus, they may be at a greater future risk of CVD. Conclusions: In the SCCS participants, we found a strong relation between exposures to high ambient levels of PM_2.5_ and self-reported CVD at enrollment.

## 1. Introduction

The U.S. Clean Air Act requires the EPA to set National Ambient Air Quality Standards for “criteria air pollutants,” including carbon monoxide, lead, ground-level ozone, nitrogen dioxide, sulfur dioxide, and particulate matter. These pollutants can cause property damage and harm the environment and an individual’s health. Aerosolized particulate matter pollution, especially fine inhalable particulates with diameters of 2.5 micrometers or smaller (PM_2.5_), may pose the most considerable cardiovascular health risks globally. PM_2.5_ is directly emitted from sources such as transport and industrial combustion, manufacturing processes, construction sites, unpaved roads, agricultural fields, smokestacks, or fires. Others form in the atmosphere because of chemical reactions from the pollutants emitted from power plants, industries, and automobiles. Exposure to PM_2.5_ is associated with a variety of health problems, including premature death in people with heart or lung disease, nonfatal heart attacks, an irregular heartbeat, diabetes, stroke, and decreased lung function [1,2]. People with heart or lung diseases, children, and older adults are the most vulnerable to airborne particulate exposures. The mortality from cardiovascular disease (CVD) such as stroke, diabetes, heart attack, and many other chronic illnesses tend to be higher among Black people than other populations in the United States [3].

Studies reporting the association between ambient fine particulate matter air pollution and adverse CVD outcomes have been inconsistent. The American Heart Association has argued that the relationship between PM_2.5_ and CVD and mortality may be the result of systemic inflammation and endothelial dysfunction [4]. Recent work has found that exposure to PM_2.5_ adversely impacts cardiovascular health [5]. Wellenius et al. (2012), using a time-stratified case-crossover study design, assessed the risk of ischemic stroke onset and PM_2.5_ concentrations in the hours and days preceding each event and found that the likelihood of stroke in 24 h of moderate PM_2.5_ exposure (15–40 μg/m^3^) was 34% higher than for periods classified as “good” (≤15 μg/m^3^) [6].

In contrast, a study on stroke found that short-term changes in PM_2.5_ exposures did not lead to increased risks of hospital admissions for stroke or cerebrovascular disease [7]. Methodological considerations have impacted the ability to categorize the limited studies on PM_2.5_ and CVD. For example, Yuan et al. (2019), in a recent meta-analysis of 16 cohort studies around the world, found significant relationships between PM_2.5_ and CVD incidence in North America and Europe but not in Asia [8]. They highlight the numerous methodological issues that may bias assessments of this relationship, including population characteristics, exposure measurement, covariable adjustments, and definitions of CVD. Similarly, a previous meta-analysis documented similar limitations with some studies relying on small samples [9], while other studies only have access to ground level estimates of PM_2.5_ exposures in urban areas.

The Southern Community Cohort Study (SCCS) allows us to test the association of long-term exposures of PM_2.5_ on self-reported CVD at enrollment across a large and diverse geographic region of the United States [10]. Furthermore, the SCCS offers an opportunity to understand the effects of air pollution on older adults’ health. Our analytic cohort comprised roughly 67% Black and 33% White participants, with comparable socioeconomic backgrounds. Thus, this sample composition allows us to consider racial disparities without the usual confounding effect of SES associated with national samples. Our ability to integrate remotely sensed MODIS satellite PM_2.5_ exposure data with U.S. EPA ground air monitoring data allowed us to characterize PM_2.5_ exposures across rural and urban locations in the entire region at a relatively high spatial resolution [11]. Specifically, we examined whether SCCS participants who lived in locations with ambient PM_2.5_ concentrations above the EPA health safety criterion (annual average concentration: 12 μg/m^3^) at enrollment were more likely to report CVD at enrollment than participants living in locations with lower concentrations of PM_2.5_.

## 2. Materials and Methods

### 2.1. Study Sample

The SCCS recruited enrollees from 12 states spread across the southeast. These states are Alabama, Arkansas, Florida, Georgia, Kentucky, Louisiana, Mississippi, North Carolina, South Carolina, Tennessee, Virginia, and West Virginia. Participants, thus, offer a unique opportunity to follow a cohort of low-income Black and White residents of the American southeast who were 40–79 years of age at enrollment. One of the original goals of the SCCS was to “elucidate and discover the sources of higher rates of cancer and other diseases among African Americans in comparison with Whites in this region” [12].

The SCCS recruitment began in March 2002 and ended in September 2009. Enrollment of most participants (about 85%) occurred at community health centers (CHCs). CHCs are primary care clinics that serve populations with limited access to health care in urban and rural settings, which offer essential health and preventive services primarily to low-income residents [10]. A total of 68 CHCs participated in the SCCS.

We selected a subsample of 44,610 SCCS participants to include in this study. Participants who

1. self-reported Black or White race,
2. were recruited through a CHC, and
3. we were able to model daily measures of PM_2.5_ exposure from ambient and satellite captured data for twelve months before their enrollment composed our analytic cohort.

This analytic cohort allows us to compare Black and White people with similar air pollution and PM_2.5_ exposure scenarios.

### 2.2. Enrollment Instrument

At enrollment, participants completed a 45–60 min, in-person interview administered by CHC personnel specially trained by the SCCS staff to recruit participants and administer the baseline questionnaire. The questionnaire collected information about various participant social characteristics (e.g., education, marital status, household income, work status, age, sex, race), behaviors (e.g., tobacco use), health status (e.g., medical history, family medical history, medication use, emotional well-being), and living environment (e.g., residence location, and air quality inside of the home).

The individual participant social characteristics included in our analyses were education (less than high school, high school graduate, vocational/technical/some college, college graduate or professional education), marital status (married or with a partner, divorced, widowed, single), household income (less than USD 15,000, USD 15,000–24,999, USD 25,000–49,999, equal to or greater than USD 50,000), work status (employed or not employed), age (less than 65 years or 65 years and older), sex (male or female), race (Black or White). We also included a self-report of air quality inside the home (poor, fair, good, excellent).

We created a cardiovascular disease risk variable (CVDRisk) for each participant based on their medical history. Cardiovascular risk refers to risk factors that increase the likelihood of experiencing cardiovascular events or developing diabetes [13]. Thus, for those participants who reported at enrollment that they had hypercholesterolemia, hypertension, or a history of smoking, we identified them as at risk of cardiovascular disease.

### 2.3. Air Pollution Exposure

We used a novel estimate of aerodynamic PM_2.5_ exposure that integrated ground-level measurements with satellite measurements of the total amount of aerosols within the air column (represented by aerosol optical depth (AOD)) from the Moderate Resolution Imaging Spectroradiometer (MODIS) instrument onboard the NASA Aqua satellite [11,14]. A major challenge in studying the relationship between air pollution and health outcomes is the characterization of exposures at the individual level. The exposure measurements typically estimated rely on ground-based meteorological and ambient air monitors, which provide the best characterization of heat and pollutant concentration levels at a specific location and time. However, considerable temporal and spatial gaps in these data limit efforts to understand health outcomes in non-metropolitan locations because air quality monitoring stations are strategically placed in high population density areas in the United States. Furthermore, fixed site stations vary in the frequency of measurement from hourly to once every few days. Our data overcome these limitations through the integration of remote sensing satellite data with ground monitoring station data. Estimated daily spatial surfaces of ground-level PM_2.5_ concentrations using the B-spline and inverse distance weighting (IDW) surface fitting techniques leveraging the National Aeronautics and Space Administration (NASA) Moderate Resolution Imaging Spectrometer (MODIS) data to complement U.S. Environmental Protection Agency (EPA) ground observation data of ambient PM_2.5_ for the years prior, during SCCS enrollment, and through to the present. MODIS instruments on the Terra and Aqua satellites provide a measure of aerosol optical depth (AOD), which is the measure of the degree to which sunlight is scattered and absorbed by aerosols of various sizes throughout the entire atmospheric column. MODIS observations are available for any area up to two times each day. Hazard data were processed to derive the surrogate PM_2.5_ estimates. As we have demonstrated elsewhere, integrating satellite remote sensing data with ground monitoring station data yields better estimates of exposure than either source alone [11,15] for health studies across a wide geographic region by providing a more complete daily representation of PM_2.5_ and reduces the errors in the PM_2.5_-estimated surfaces. Furthermore, although the IDW technique can introduce numerical artifacts that could be due to its interpolating nature, which assumes that maxima and minima can occur only at the observation points, the daily IDW PM_2.5_ surfaces had smaller errors in general with respect to observations, than those of the B-Spline surfaces.

Daily measures of PM_2.5_ were collected using a spatially and temporally continuous grid of 3-kilometer cells across the 12-state region. For each participant, the residence at enrollment was assigned to a 3-kilometer grid cell to protect their identities (an approach that was required under IRB) and used to associate individual exposures. For these analyses, we estimated the annual average daily exposure for each participant based on their date of enrollment into the SCCS. Thus, each participant’s annual average daily residential exposure was calculated using daily measures within a 3-kilometer grid cell surrounding their location for the twelve months prior to enrollment from the date of enrollment, when all other data from the patient enrollment questionnaire were collected.

We used the EPA National Ambient Air Quality Standards, which identified a threshold for unhealthy air of fine particulate matter (PM_2.5_) at an annual average level of 12.0 μg/m [16]. We created a PM_2.5_ unhealthy exposure indicator variable of PM_2.5_ over 12 μg/m^3^ based on each SCCS participant’s residential location.

### 2.4. Self-Report Cardiovascular Disease Ascertainment

The enrollment questionnaire asked participants about their medical histories. Specifically, it asked the following three questions regarding CVD: (1) “Has a doctor ever told you that you have had a stroke, mini-stroke, or transient ischemic attack (TIA)?”, (2) “Has a doctor ever told you that you have had diabetes?”, (3) “Has a doctor ever told you that you have had a heart attack or coronary artery bypass surgery?” Participants could respond to these questions with “Yes”, “No”, or “I Don’t know”. We modeled responses for those who responded either “Yes” or “No” to these questions.

Self-reports on medical conditions that are well defined and easily diagnosed show high positive predictive value compared to conditions that exhibit complex symptoms. For example, strokes can exhibit well-recognized motor impairments and more subtle symptoms that may diminish the individual’s ability to self-report a stroke accurately, if not previously identified by a physician. Thus, the SCCS participants may have under-reported strokes because of less access to care or vague symptoms or silent strokes not detected by the patient or their physician. However, elderly Black people have exhibited a higher sensitivity of stroke reports than White people [17]. Similarly, some participants may have under-reported diabetes or heart attacks due to less access to care. Despite these limitations, for epidemiological research, self-reports such as those used in the SCCS are regarded as a valid tool [18].

### 2.5. Statistical Analyses

Our study included 44,610 SCCS participants recruited from 68 CHCs across 12 southeastern States. To account for the nesting of data at two levels (e.g., state and health center), we modeled the response to whether the participant reported CVD using a multilevel logistic regression model. Clustering introduces within-cluster homogeneity, which can be addressed using this type of modeling [19]. When data have a multilevel structure, participants within the same cluster (e.g., CHC) may have responses or outcomes correlated with one another due to their common context. For example, each CHC may offer a distinct level of services, staff skills, or access to technology, and its catchment service area may differ as well (e.g., neighborhood poverty level). Thus, participants from the same CHC may be more similar than participants from different CHCs. Similarly, participants from the same state may experience different state-level contexts than those from different states. Each state has its environmental regulations, zoning, other policies, and program eligibility (i.e., Medicaid); while all participants from a particular state share the same context, those in other states experience different contexts. Multilevel modeling incorporates cluster-specific random parameters that account for the dependency of the data by partitioning the total individual variance into variation due to clusters and the individual-level variation. Failure to account for the intra-cluster correlation falsely inflates the precision of the estimates [20].

Our estimates are based on the 44,610 participants who had complete data for our model specification using a binomial probability distribution and a logit link function. We present fixed effects for the environmental, social, and biological characteristics of the participants given our research question of interest and the role of participant characteristics. Therefore, we use random intercepts for state and community health center levels of data clustering.

## 3. Results

At enrollment, 83 percent of the participants lived in locations exposed to unhealthy levels of PM_2.5_ (Table 1). Only 10 percent of the participants were over the age of 65 years, but only 36.3 percent were employed. This low level of employment may be the result of considerable illness burden, with 70 percent of the participants reporting risk for cardiovascular disease. Almost one third (30.4%) of the participants had less than a high school education and another third (35.9%) had only a high school education.

**Figure 1.**
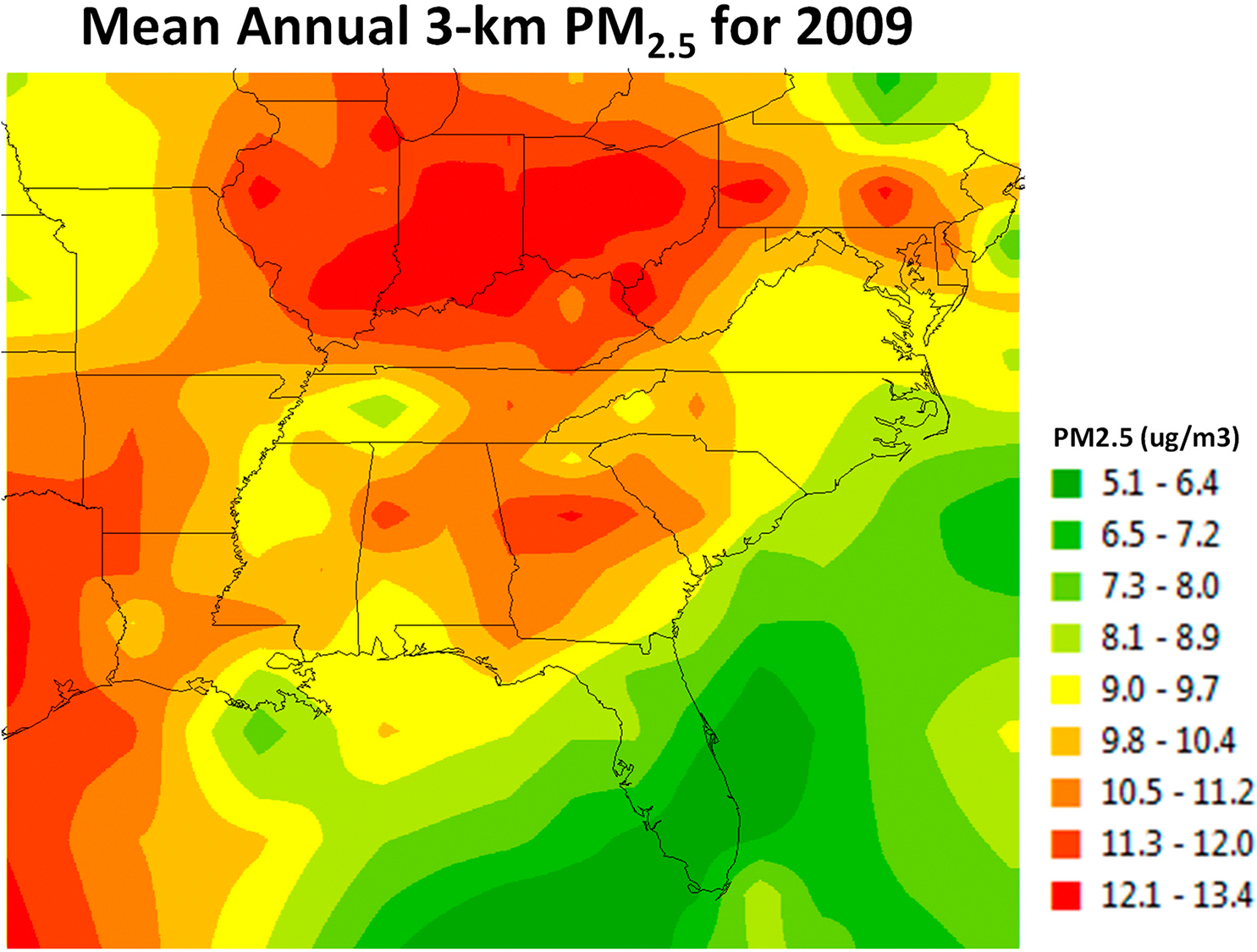
Southern Community Cohort Study and Average Annual PM_2.5_ Exposures, 2009.

Figure 1 illustrates the annual average PM_2.5_ concentration levels for the 12-state region where all the participants in the Southern Community Cohort Study resided in 2009. The average annual PM_2.5_ concentrations vary considerably over the 12-state Southern Region of the United States. With the highest concentration levels observed in the northern parts of Georgia, Mississippi, and Alabama, along with various specific locations in many of the other study region states (e.g., Tennessee, Kentucky, and the Carolinas).

Table 2 presents the proportion of respondents who lived in locations below and above the EPA criterion standard for PM_2.5_ by their social and medical risk characteristics. The average annual exposure for those living below the EPA standard was 10.3 μg/m^3^. The average annual exposure for those living in regions where exposures exceeded the EPA unhealthy standard was 14.0 μg/m^3^ (*p* < 0.0001). Notably, a higher proportion of Black than White people were likely to live in environments with more significant PM_2.5_ exposures. Nevertheless, a higher proportion of respondents with cardiovascular risk lived in locations with lower PM_2.5_ exposures. Those living in lower exposure areas were more likely to be married, have higher incomes, report better air quality indoors, and be younger than those living in higher exposure environments.

Modeling unhealthy levels of PM_2.5_ (i.e., above 12 μg/m^3^) alone in our multilevel logistical model indicates that individuals living in environments with higher concentrations of fine particulates who report CVD is 16.5% (95% confidence interval: 7.4–26.3%) higher than those living in communities with lower exposures. Accounting for the social and biological characteristics of the participants, our multilevel logistic model (Table 3) finds individuals living in a region with PM_2.5_ exposures above the EPA ambient level criterion reporting CVD is 13.4% higher (95% confidence interval: 4.3–23.2%) than those living in areas with lower exposures. We find no difference in males and females reporting CVD, with the odds ratios for males estimated as 1.02. However, the odds ratio for Black people is 1.097 (95% confidence interval: 1.038–1.07) compared to White people reporting CVD at the time of enrollment. This 10 percent higher odds of CVD reports among Black participants is statistically significant, and clinically relevant because Black people have high levels of clinical risk factors and are more likely to live in locations with higher PM_2.5_ concentrations. Our odds ratio estimate for our CVD risk variable is 4.85, indicating that those with a history of hypertension, hypercholesteremia, or smoking have an increased odds of reporting a CVD at enrollment of 385 percent, compared to those without such medical history.

We find no random effect at the state level (Z = 1.16, sig. 0.25) but a significant random effect at the CHC level (Z = 4.08, sig. 0.023). This can be further observed in the interclass correlations for CVD at the state level (0.0042) and the CHC level (0.023). Thus, participants within the same CHC are more similar than if they had been selected completely at random. Accounting for these similarities within the clinic level improves our estimates of fixed effects for variables of interest, such as exposure to PM_2.5_ (Table 4).

## 4. Discussion

Our findings indicate that participants in the SCCS at enrollment report 13.4% more CVD at enrollment if they lived in areas with moderate to high ambient concentrations of PM_2.5_, considered unhealthy by the Environmental Protection Agency. After accounting for social characteristics and medical history, no differences in self-reported CVD for male and female participants are observed. However, Black people may be at higher risk of CVD [21], about 10% higher odds after accounting for social characteristics and medical history. Black and White people in the SCCS were recruited through Community Health Centers; therefore, they share common SES and other characteristics of low-income populations (e.g., high disease burden) that allow for racial comparisons that are less confounded than typical national estimates of racial health disparities. Genomic sequencing studies provide additional evidence that Black people are at a greater risk of CVD due to polymorphisms in microRNA genes [22]. Interactions between polymorphisms in genes that govern endothelial function, cigarette smoking and risk of CVD has been reported in Black People [23]. Taken together, these studies emphasize that interplay between environmental factors, of which PM_2.5_ is one, and genetics exacerbate the CVD risk in Black people.

As we previously reported [24], many SCCS participants were at risk of cardiovascular disease due to high reports of the following clinical risk factors: hypertension (56%), hypercholesterolemia (35%), and a history of smoking currently or in the past (65%). Even though a higher percentage of the at-risk participants lived in areas with lower concentrations of PM_2.5_, being at clinical risk increased the odds of reporting a history of CVD at enrollment by 385%. Poor health behaviors, such as poor diets and smoking, lead to an increased biological risk that plays a major role in the development of cardiovascular disease in all environments.

Exposure to chemical stressors such as aerosolized fine particulate matter and other associated chemical stressors (e.g., polycyclic aromatic hydrocarbons, metals, and traffic-related pollution) are strongly associated with increased morbidity and premature death from CVD in exposed adults and those already suffering from a wide range of illnesses [25]. While this is particularly evident in environmentally compromised communities, very little remains known about exactly how non-chemical exposures from social environmental factors interact with the effects of chemical exposures on CVD health outcomes.

It is important to recall that within the SCCS we are examining Black and White participants where socioeconomic differences are minimal [10]. However, reported disease prevalence may reflect disparities in previous access to care or treatment differences in clinical settings as the SCCS assessed CVD relying on self-reports based on previous medical care. Nevertheless, we find an association between unhealthy PM_2.5_ concentration exposures and CVD reports in this cohort, accounting for social characteristics and medical history.

The southeastern U.S. has experienced a 48% decrease in the annual, seasonally adjusted, regional average concentration of PM_2.5_ from 2000 to 2017. This decrease in PM_2.5_ began before the start of the SCCS and has steadily continued throughout the study [26]. However, the annual regional average was above the EPA’s unhealthy criterion (over 12 μg/m^3^) before the SCCS enrollment began and until well after the SCCS enrollment ended. In fact, the decline in PM_2.5_ from 2000 to 2017 could be because the EPA has strengthened the 24-hour PM_2.5_ standard by revising the level from the previous level of 65 to 35 μg/m^3^ as of October 2006 [27]. In 2005, the World Health Organization issued a global recommendation for unhealthy levels PM_2.5_ exposure to over 10 μg/m^3^, a level regularly surpassed in many parts of the United States and the world. Our study highlights the utility of setting PM_2.5_ standards that can be used to regulate the harmful health effects of air pollution.

While assessing the relationship between PM_2.5_ and CVD, the PM source should be considered. The present study captured the levels of overall PM_2.5_ mass concentrations, but not in a source-specific manner. Aside from automobile emissions, a strong association between fossil-fuel-derived PM_2.5_ (especially from combustion sources such as coal burning) and CVD has been reported [25,28].

Another possibility for the relationship between PM_2.5_ exposure and CVD could be due to the different toxicities of individual components of PM_2.5_ versus the total concentration levels [29]. The limited toxicological evidence related to the chronic effects of PM_2.5_ handicaps our understanding of how the individual components of PM_2.5_ drives health effects such as diabetes, heart attack, and stroke [30]. Employing biomarkers from the repository of well-established cohorts such as SCCS could increase our understanding of the biochemical and molecular underpinnings of air pollution-associated CVD. Some of the various biomarkers (conventional as well as omics-related) employed for CVD are discussed in detail by Juarez et al. (2020) [31]. These biomonitoring data could complement the ambient PM_2.5_ data to provide a comprehensive assessment of the association between PM_2.5_ exposure and CVD.

The SCCS allowed us to examine long term exposures because participants have lived in their enrollment residence for, on average, ten years. While rural poor people may move, they generally move within a limited geographic range, usually within the same community [32]. Thus, we can conclude that we find an association in long-term exposures to unhealthy levels of PM_2.5_ and self-reported CVD at enrollment for SCCS participants that represent a large and diverse geographic region of the United States.

## Figures and Tables

**Table 1.** Southern Community Cohort Study Participant Characteristics Enrolled from Community Health Clinics in 12 Southern States (2002–2009) (*n* = 4460).

Variable		Percent

Over 12 ug/m^3^ PM_2.5_		83.0
Male		39.1
Black		67.1
Employed		36.3
Senior		10.1
	Education	
No High School		30.4
High School		35.9
Some College		24.3
College or more		9.4
	Marital Status	
Married		32.4
Separated		35.1
Widowed		9.6
Single		22.8
	Income	
Less than USD 15,000		60.0
USD 15,000–25,000		21.7
USD 25,000–50,000		12.7
More than USD 50,000		5.7
	Inside Home Air Quality	
Poor		6.1
Fair		28.8
Good		52.2
Excellent		12.9
	Cardio-Metabolic Disease Risk	
CMD Risk		70.2

SCCS participants were recruited from Alabama, Arkansas, Florida, Georgia, Kentucky, Louisiana, Mississippi, North Carolina, South Carolina, Tennessee, Virginia, and West Virginia.

**Table 2.** Percentage of Participants by Social Characteristic Living in Low and High PM_2.5_ Exposure Locations in 12 Southeastern United States.

Variable	PM_2.5_ < 12 μg/m^3^	PM_2.5_ > 12 μg/m^3^	*p*-Value

Education *			0.799
Less than High School	30.2%	30.4%	
High School	36.4%	35.8%	
Voc., Tech, or Some College	24.0%	24.3%	
College Graduate or Beyond	9.3%	9.5%	
Marital Status *			<0.0001
Married or with Partner	34.5%	32.0%	
Divorced	34.5%	35.2%	
Widowed	10.2%	9.5%	
Single	20.7%	23.3%	
Household Income *			<0.0001
Less than USD 15,000	59.1%	60.2%	
USD 15,000–24,999	19.9%	22.0%	
USD 25,000–49,999	13.5%	12.5%	
USD 50,000 or more	7.5%	5.3%	
Air Quality Inside *			<0.0001
Poor	5.6%	6.1%	
Fair	26.4%	29.3%	
Good	52.2%	52.2%	
Excellent	15.8%	12.3%	
Employed *	36.7%	36.2%	0.414
Elder (>65 years old) *	12.2%	9.7%	<0.0001
CVD Risk (Present) *	70.6%	70.1%	<0.385
Race—Black *	65.1%	67.5%	<0.0001
Sex—Male *	39.2%	39.0%	0.737
PM_2.5_ More 12 μg/m^3^ **	10.3 μg/m^3^	14.0 μg/m^3^	<0.0001

* Chi-square test;

** Mean exposure level of PM_2.5_.

**Table 3.** Multilevel Logistic Regression Model Estimated Fixed Effects for Reported Cardiovascular Disease at Enrollment by SCCS Participants.

		Estimated Fixed Effects					

Model Term	Coefficient	SE	t	*p*-Value	OR*	Lower 95% CI for OR	Upper 95% CI for OR

Intercept	−1.940	0.0848	−22.885	<0.001	0.144	0.122	0.170
			PM_2.5_				
>12 μg/m^3^	0.125	0.0425	2.950	0.003	1.134	1.043	1.232
<12 μg/m^3^	Ref						
			Sex				
Male	0.021	0.0237	0.879	0.379	1.021	0.975	1.070
Female	Ref						
			Race				
Black	0.092	0.0281	3.287	0.001	1.097	1.038	1.159
White	Ref						
			Employed				
Yes	−0.593	0.0260	−22.816	<0.001	0.553	0.525	0.581
No	Ref						
			Age				
65 or Older	0.276	0.0357	7.747	<0.001	1.318	1.229	1.414
64 or Younger	Ref						
			Education				
College+	−0.144	0.0462	−3.120	0.002	0.866	0.791	0.948
Some College	−0.085	0.0315	−2.692	0.007	0.919	0.864	0.977
High School	−0.149	0.0274	−5.443	<0.001	0.861	0.816	0.909
No High School	Ref						
			Marital Status				
Single	−0.259	0.0610	−6.956	<0.001	0.654	0.581	0.737
Widowed	0.166	0.0386	−3.639	<0.001	0.869	0.806	0.937
Separated	−0.079	0.0283	−3.569	0.005	0.901	0.850	0.954
Married	Ref						
			Income				
>USD 50,000	−0.424	0.0610	−6.956	<0.001	0.654	0.581	0.737
USD 25,000–50,000	−0.140	0.0386	−3.639	<0.001	0.869	0.806	0.937
USD 15,000–25,000	−0.105	0.0293	−3.569	<0.001	0.901	0.850	0.954
<USD 15,000	Ref						
			Air Quality Inside				
Excellent	0.158	0.0553	2.859	0.004	1.171	1.051	1.305
Good	0.049	0.0483	1.014	0.311	1.050	0.955	1.155
Fair	0.031	0.0500	0.622	0.534	1.032	0.935	1.138
Poor	Ref						
			CVD Risk				
Present	1.580	0.0320	49.322	<0.001	4.854	4.558	5.168
Absent	Ref						

		**Estimated Random Effects**					
	**Estimate**	**SE**	**Z**	***p*-Value**	**Lower**	**Upper**	
Variance of STATE	0.014	0.012	1.160	0.246	0.003	0.076	
Variance of Clinic nested in STATE	0.062	0.015	4.077	0.000	0.039	0.101	

Probability distribution: Binomial. Link function: Logit. OR

* Odds Ratio.

**Table 4.** Interclass Correlation for CVD.

	PM_2.5_ Only (Univariate Model Not Shown)	Multiple Variables with PM_2.5_ (Table 3)

CVD ICC State Level	0.003499697	0.004159402
CVD ICC State(Clinic)	0.040538159	0.022579613

## Data Availability

The data used in this study are publicly available upon request from the Southern Community Cohort Study and the Meharry Medical College Center of Excellence in Health Disparities Exposome Collaborative by contacting Paul Juarez (pjuarez@mmc.edu).
